# Survey on fan-beam computed tomography for radiotherapy: Current implementation and future perspectives of motion management and surface guidance devices

**DOI:** 10.1016/j.phro.2023.100523

**Published:** 2023-12-10

**Authors:** Chrysi Papalazarou, Sima Qamhiyeh, Robert Kaatee, Joke De Rouck, Esther Decabooter, Guido C. Hilgers, Koen Salvo, Jacobus van Wingerden, Hilde Bosmans, Brent van der Heyden, Geert Pittomvils, Evelien Bogaert

**Affiliations:** a Leiden University Medical Center, Leiden, the Netherlands; b Department of Radiation Oncology, University Hospitals Leuven, Leuven, Belgium; c Radiotherapy Institute Friesland, Leeuwarden, the Netherlands; d Department of Radiotherapy, AZ Sint Lucas, Ghent, Belgium; e Department of Radiation Oncology (Maastro Clinic), GROW School for Oncology, Maastricht University Medical Centre+, Maastricht, the Netherlands; f Radiotherapiegroep, Arnhem/Deventer, the Netherlands; g Department of Radiotherapy, AZ Sint-Maarten, Mechelen, Belgium; h Department of Medical Physics, Haaglanden Medical Centre, Leidschendam, the Netherlands; i Department of Radiology, University Hospital Gasthuisberg, Leuven, Belgium; j Medical Physics and Quality Assessment, Department of Imaging and Pathology, KULeuven, Leuven, Belgium; k Department of Oncology, Laboratory of Experimental Radiotherapy, KU Leuven, Leuven, Belgium; l IBiTech-MEDISIP, Department of Electronics and Information Systems, Ghent University, Ghent, Belgium; m Department of Radiation-Oncology, Ghent University Hospital, Ghent, Belgium

**Keywords:** Radiotherapy, Computed tomography, Quality assurance, Survey, 4DCT, Surface guidance

## Abstract

**Background and purpose:**

This work reports on the results of a survey performed on the use of computed tomography (CT) imaging for motion management, surface guidance devices, and their quality assurance (QA). Additionally, it details the collected user insights regarding professional needs in CT for radiotherapy. The purpose of the survey is to understand current practice, professional needs and future directions in the field of fan-beam CT in radiation therapy (RT).

**Materials and methods:**

An online institutional survey was conducted between 1-Sep-2022 and 10-Oct-2022 among medical physics experts at Belgian and Dutch radiotherapy institutions, to assess the current status, challenges, and future directions of motion management and surface image-guided radiotherapy. The survey consisted of a maximum of 143 questions, with the exact number depending on participants’ responses.

**Results:**

The response rate was 66 % (31/47). Respiratory management was reported as standard practice in all but one institution; surface imaging during CT-simulation was reported in ten institutions. QA procedures are applied with varying frequencies and methodologies, primarily with commercial anatomy-like phantoms. Surface guidance users report employing commercial static and dynamic phantoms. Four main subjects are considered clinically important by the respondents: surface guidance, CT protocol optimisation, implementing gated imaging (4DCT, breath-hold), and a tattoo-less workflow.

**Conclusions:**

The survey highlights the scattered pattern of QA procedures for respiratory motion management, indicating the need for well-defined, unambiguous, and practicable guidelines. Surface guidance is considered one of the most important techniques that should be implemented in the clinical radiotherapy simulation workflow.

## 1 Introduction

The importance of respiratory motion management has been established for thoracic and abdominal tumours [1], while it is considered mandatory in stereotactic body radiation therapy (SBRT) for peripheral lung tumours [2]. Its use in routine clinical practice has increased, and optimal tumour targeting in sites affected by respiratory motion has become more relevant with growing numbers of hypofractionated radiotherapy in these treatment sites [3], [4]. A large recent AAPM respiratory motion management survey by Ball et al. [1] showed that the three most frequently used simulation methods for thoracic and abdominal patients were four-dimensional Computed Tomography (4DCT, 93 %), followed by breath-hold (51 %) and free-breathing (31 %) three-dimensional CT (3DCT). De Oliveira Duarte et al. [5] conducted a national survey in France (2019) solely focusing on 4DCT planning and found high scan repetition rates either to check the reproducibility of the respiratory movement or due to the presence of image artefacts. 4DCT imaging is continuously evolving with new techniques, such as new sequential scanning modes [6] aiming at artefact reduction. An update of the guidelines on best practices is necessary to keep up with these advancements.

Surface Guided Radiotherapy (SGRT) describes the integration of Surface Imaging (SI) into the radiotherapy workflow [7] and has been adopted for patient setup, monitoring and gating [8]. The growing interest in SGRT is reflected in the increasing number of related publications [9], [10] and worldwide installations [11]. Batista et al. [8] and Padilla et al. [7] have previously reported on surveys conducted on behalf of ESTRO and AAPM, which focused on clinical applications, Quality Assurance (QA) programs, and the need for formal guidance for SGRT. SI equipment is mostly connected to linac delivery-systems for controlled beam interruptions when a motion threshold is exceeded. Although similar installations with surface guidance are possible in the CT-simulation-room [12], a limited number of institutions has been reported on this specific use (16 % and 34 % of the institutions in [8] and in [7], respectively). Padilla et al. [7] concluded that despite the rapidly improving technology and the widespread use of SI systems, the absence of guidance on integration in the clinical environment is causing delays in its implementation.

Current guidelines on the QA of fan-beam CT in radiation oncology are relatively old [13], [14], [15]. To obtain an understanding of current practice, professional needs and future directions in this field, it was considered advisable to conduct a survey with a broad scope. First, the survey questioned currently installed hardware and clinical practices regarding CT imaging for simulation, delineation and dose calculation. A further part of the survey enquired on practices in respiratory motion management and surface guidance. Finally, users’ insights and preferences on future developments in the field were collected. The first part of the findings of the survey is detailed in [16]. In this article, we report on the second part, while putting it into perspective of the vision expressed by the respondents.

## 2 Materials and methods

In 2021, the Netherlands Commission on Radiation Dosimetry (Nederlandse Commissie voor Stralingsdosimetrie, NCS) called for a task group to formulate updated guidelines on the QA of fan-beam CT in radiation oncology. An online questionnaire was provided between September 1st and October 10th, 2022, to all Belgian and Dutch radiotherapy institutions. Medical Physics Experts in radiotherapy (MPE RT) of 47 institutions were invited by email to provide one answer per institution. Anonymous completion was an available option, if desired.

The subsections of the survey covered the following subjects: technical specifications of the CT scanner(s), QA, simulation workflow, imaging for dose calculation, motion management during CT acquisition, surface guided imaging, and the respondents’ vision on CT imaging for radiotherapy. A maximum of 143 questions, incorporated in a Microsoft Teams (Microsoft Corp., Redmond, Washington) environment, could be posed to the respondents, where the exact number was determined dynamically based on the respondents’ input. The terminology defined in the survey and used throughout this paper includes the abbreviations: MPA (Medical Physics Assistant), MPE RT (Medical Physics Expert Radiotherapy), MPE RD (Medical Physics Expert Radiology), RTT (Radiotherapy technologist), RO (radiation oncologist) and PA (Physician Assistant).

The data was reviewed to improve data quality, i.e., to avoid duplicate, inconsistent, or contradictory answers within the same institution. When anomalies were suspected in the reported hardware characteristics, vendors were contacted for verification. Microsoft Excel (Microsoft Corporation, 2018, USA) and IBM SPSS Statistics for Windows, version 28 (IBM Corp., Armonk, N.Y., USA) have been used for numerical and statistical analysis. Friedmann non-parametric statistical test for comparing mean ranks of ordinal data has been applied in SPSS.

## 3 Results

A total of 31 institutions replied to the survey, giving a response rate of 66 %. The affiliation of the respondents was reported to be a university hospital by 42 %, non-university hospital by 48 % and unspecified by 10 %.

**Fig. 1:**
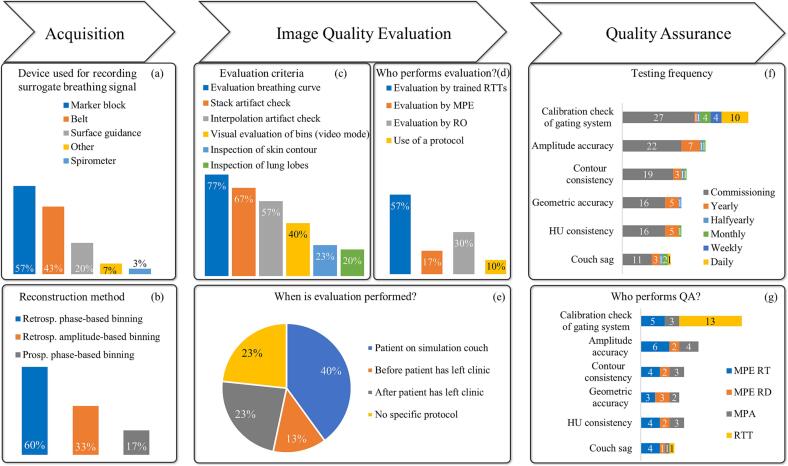
Overview of the responses regarding the application of respiratory motion management in CT. Bar charts have been used for questions where multiple replies were allowed, hence the sum of percentages may be >100 %. Percentages have been calculated with a denominator of N = 30. Absolute units instead of percentages have been used in (f) and (g) due to the low numbers.

Some form of respiratory management (breathing-correlated 4DCT or breath-hold) is standard practice in all but one institution (30/31), hence reported percentages for the related questions are computed with a denominator of N = 30. Fig. 1 summarizes the main findings of the survey regarding respiratory motion management. Different devices are employed to capture the surrogate respiratory signal, including infrared marker block (57 %), belt (43 %), and surface guidance (20 %); one institute uses a spirometer. Most respondents use retrospective phase-based (60 %) or amplitude-based (33 %) binning; a few users (17 %) also report the option of prospective phase-based binning. A ten-bin reconstruction is the most common (87 %). Deep inspiration breath-hold is employed in most institutions (63 %). These techniques may be combined in some institutions, as multiple answers were allowed for this question.

The majority of respondents (77 %) systematically performs checks on the reconstructed gated/4D images. The items checked vary, although in most cases more than one criterion is employed, see Fig. 1(c). During these checks, the patient remains on the CT couch or in the department in 40 % or 13 % of institutions, respectively. In all but three institutions, there is no fixed protocol for these checks, but the expertise of either RTTs or RO/MPE is relied upon.

A calibration check of the gating device is typically performed as part of commissioning (27/30), or less frequently as part of daily (10/30), weekly (4/30), or monthly (4/30) QA. Contour consistency (19/30), geometric accuracy (16/30), HU consistency (16/30), and couch sagging (11/30) are often checked on commissioning but are included less and with varying frequencies in routine QA; see Fig. 1(f). Daily calibration checks are performed predominantly by RTTs, while the remaining listed QA tasks are performed varyingly by MPEs RT, MPEs RD, and MPAs; see Fig. 1(g) for the distribution of tasks.

**Fig. 2:**
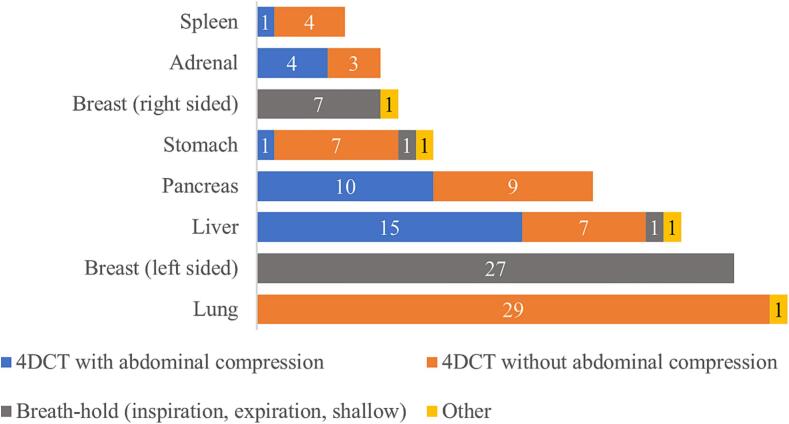
Responses on the clinical indications for which respiratory motion management is applied in the CT room (N = 30 institutions).

Main indications for breath-hold or 4DCT, detailed in Fig. 2, are lungs (100 %), left-sided breast (90 %), and liver (80 %). Additional indications, reported on top of the options given in the survey, include 4DCT (with or without compression) for oesophageal tumours (33 %) and mediastinal lymphomas (13 %).

**Fig. 3:**
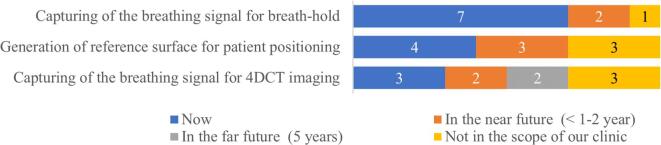
Responses regarding current and planned clinical applications of SI, of the 10 institutes that have SI systems available.

The survey also evaluated the use of SI and its planned clinical use during CT simulation in the near (within one-two years) or far (within at least five years) future, shown in Fig. 3. Surface imaging during CT-simulation is implemented in 10/31 institutions. One respondent indicated the use of SI in research and did not indicate any current clinical use. The current clinical applications include gating CT acquisition by capturing the breathing signal during breath-hold CT acquisitions (7/10) and 4DCT acquisition and reconstruction (3/10). Additionally, the generation of a reference surface for patient positioning is reported by four institutes. Meanwhile, the use of SI in collision detection was only foreseen by one respondent and was not considered in the scope of clinical practice by the remaining nine institutions.

QA procedures were applied with varying frequency and methodology. The phantoms used were mainly static with either a geometrical (8/10) or anatomy-like design (3/10). A smaller number of institutions (2/10) uses dynamic phantoms for surface guidance-based motion management strategy testing, while one institute mentions using volunteers for testing the sensitivity and range of the camera and determining signal reproducibility. All phantoms referenced by the respondents were commercially acquired. The phantoms used for testing the surface guidance system at the level of simulation are specific to CT-simulation in 50 % of the replies. Moreover, the regulation of lighting conditions in the CT room during installation and use of SI systems was taken into consideration by 50 % of the respondents.

**Fig. 4:**
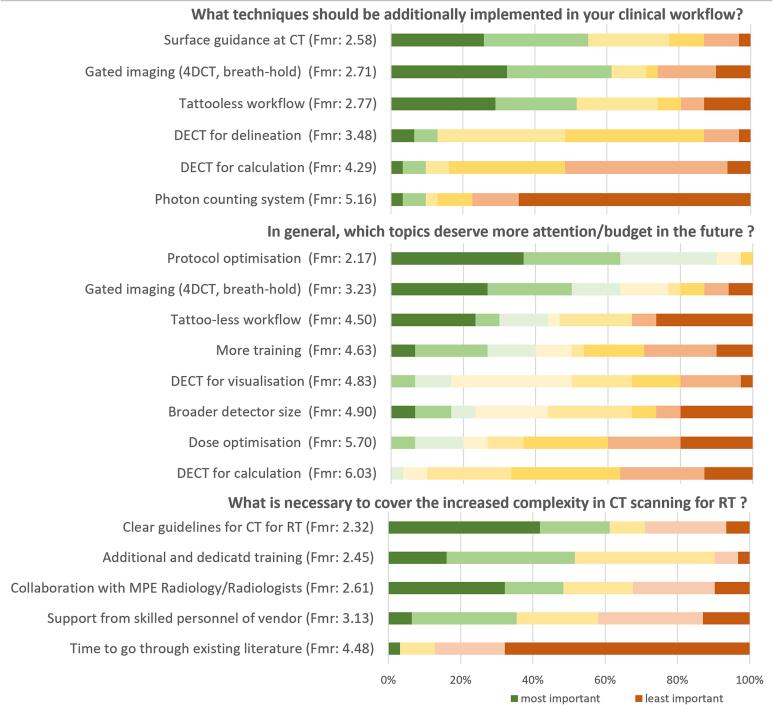
Friedman ranked answers upon questions assessing professional needs for CT in RT in Dutch and Belgian institutions. Fmr = Friedman mean rank score. Lower Fmr values relate to higher score of importance.

Regarding the professional needs of Belgian and Dutch MPEs, three specific questions were posed: (1) additional techniques that respondents wished to see implemented in their clinic; (2) actions needed to tackle the increased complexity in the field of CT imaging for radiotherapy; and (3) topics deserving more attention or financial resources in the future. A Friedman statistical analysis on the first and second level ranking questions, revealed four prevailing topics, namely ‘surface guidance at CT’, ‘optimisation of CT scan protocols’, ‘gated imaging (4DCT, breath-hold)’ and ‘tattoo-less workflow’ (significance < 0.05 with regard to the next ranked topics). Friedman mean ranks (Fmr) are given in Fig. 4, where lower values relate to higher score of importance.

Spectral imaging such as Dual-Energy CT (DECT) was given a median score of 7/10 (quartiles range: 5–7.5) in the question whether it will in future become the standard for CT-scanners in radiotherapy, at least for some indications DECT for segmentation and calculation share a fifth place only at the level 2 ranking question and with a substantially lower Friedman mean rank. DECT to improve dose calculation was ranked lower than the DECT to potentially achieve better image quality for delineation at the level 1 ranking question. Spectral imaging by photon counting, as described in [17] has been scored as not widely reachable for application. CT dose optimisation was not seen as a critical topic for the future. Only a minority of the respondents (8/31) was worried about patient exposure burden related with CT scanning for radiotherapy.

The top three priority action points are: (1) Clear guidelines for fan-beam CT applications in RT, (2) Additional and dedicated training, and (3) Collaboration with MPE Radiology and/or Radiologists. It should be noted that ‘More or specialized training for operating/commissioning the CT scanner’, incorporated in the level 1 question, ranked immediately after the top items for that question, but with lower mean rank value in the Friedman analysis. The combined information stresses the importance of providing dedicated training on the setup of a CT-based simulation workflow.

## 4 Discussion

An institutional survey has been conducted by a task group mandated by the NCS to investigate the use fan-beam CT in radiotherapy. Although the survey targeted a specific geographic region (Belgium and the Netherlands), its findings may be of interest to a broader audience within the radiotherapy community. We have reported on the clinical use of motion management techniques and surface guidance, putting them into broader perspective to other prevailing professional needs in radiotherapy CT. A separate article [16] discusses clinical practices in CT simulation, delineation and dose calculation. For the purposes of the survey, respiratory motion management was defined to include 4DCT imaging, gating, breath-hold, either with or without abdominal compression. These are connected yet distinct approaches to respiratory motion management.

The reported QA checks for 4DCT and breath-hold reveal a scattered pattern of techniques and tools used among the respondents. Although most users perform checks on all 4DCT/breath-hold datasets acquired, the criteria used vary considerably, while only a few users perform routine QA on relevant functionality of the scanner, ancillary devices and related software. Recommendations for QA of gated and/or 4DCT imaging in literature are scarce. Chen et al [18] have performed a thorough analysis of the artefacts generated during helical scanning of moving objects, and their relation to imaging parameters such as pitch and slice thickness. The effect of these artefacts on clinical delineations was shown in [19] and [20], while negative effects on clinical outcomes were demonstrated for lung and liver SBRT [21], as well as in SBRT for oligometastatic colorectal cancer [22]. The report of AAPM Task Group 76 is dedicated to the management of respiratory motion and includes general recommendations for QA of gating, various breath-hold techniques and tumour tracking. However, no recommendations for QA of 4DCT are given in that report. This indicates the need for well-defined, unambiguous, and practicable QA guidelines for 4DCT and breath-hold imaging. These guidelines should encompass machine-level as well as patient-specific evaluations, particularly in relation to the quality assessment of reconstructed images and recorded respiratory signals.

The survey assayed the current and anticipated clinical use of SI in CT simulation in Dutch and Belgian institutions, reflecting both the growing interest, as well as the barriers and limitations to its implementation. A similar scope of clinical applications is observed in other reports including [9], [10], [11]. Fig. 3 indicates a wish to have SI based gating of CT acquisition by most institutions within five years. SI is often used for motion tracking and patient guidance, while additional ancillary gating systems are used for gating during image acquisition and reconstruction. Hence, improving the online connectivity between CT-scanners and SI systems should reduce the burden of redundant technology implementation and broaden the scope of use of SI during CT-simulation. Another main use of SI is generating a reference surface to enhance patient positioning and patient motion monitoring. The obstruction of the camera by positioning devices or the patient itself must be avoided in such a scenario. The use of SI should be carefully evaluated under recommended lighting conditions in the simulation room, as well as with given patient setup conditions.

In accordance with international guidelines [11], [12], all ten institutes which use SI systems in CT simulation rooms use a variety of test objects to validate their customized SI workflow. Despite the fact that SI vendors offer basic geometrical phantoms to ensure reliable performance of the installed devices, up to 50 % of the respondents reported that additional phantoms were needed, indicating that these basic phantoms are not well-suited for testing clinical workflows.

Furthermore, some respondents anticipate a tattoo-less workflow as a potential application of SI. However, to establish a reliable tattoo-less workflow, the setup accuracy and performance should be at least equivalent to that of a tattoo/laser setup in most treatment sites [9], [23]. The fact that it was not reported as a current or near-future practice in this survey suggests that there may be obstacles to overcome in its implementation.

The survey asked respondents to prioritise future developments in CT imaging for radiotherapy in a broader sense, including on the topics covered by [16]. Besides the topics of gated imaging and SI discussed above, the responses indicated that optimising CT scan protocols to improve image quality deserves most attention and budget. Meanwhile, the use of DECT or photon-counting CTs is expected to enhance image resolution and contrast-to-noise ratio, improving delineation accuracy. Additionally, these techniques would improve the determination accuracy of tissue properties needed for dose calculations, improving the precision of tissue assignment and dose calculation for Monte Carlo dose calculation algorithms, especially in proton therapy [24], [25]. However, DECT and photon counting CTs got an intermediate ranking of techniques that should be implemented in clinic. This survey outcome might be biased due to the limited number of Belgian (N = 1) and Dutch institutions (N = 3) currently providing proton therapy.

Radiation dose due to CT imaging for radiotherapy was not considered a concern (74 %), despite the fact that multiple CT scans are acquired within the framework of adaptive radiotherapy and considerable scan ranges are reported [26]. The process of protocol optimization should ensure a sufficient CT image quality allowing for accurate outlining of the treatment target and surrounding organs, whilst minimizing healthy tissue dose [27]. We interpret the survey responses as indicating the community’s view of image quality as the determining factor, for which imaging dose is a secondary consideration. This outcome is in line with the main professional needs obtained from the survey, namely optimising CT protocols, gated imaging, surface guidance, and DECT for delineation, all of which could contribute to higher treatment dose conformity and better sparing of healthy organs.

In summary, it was found that 4DCT is indispensable in current and future clinical practice. The survey discerned a clear need for additional QA guidelines regarding machine QA, as well as patient-specific QA, with emphasis on guidelines for CT image quality. Surface guidance is increasingly used during CT simulation and is indicated as an important future topic, although some hesitancy was observed in certain clinical applications. As the use of ancillary devices surrounding the CT scanner continues to expand in scope and complexity, the importance of interoperability between these devices and their integration into the clinical workflow becomes apparent. QA procedures should be designed with this aspect in mind, and careful end-to-end testing of these devices and workflows is recommended. The authors strongly encourage the development of open platforms and standardized interfaces by vendors, which enable different devices to operate and communicate with each other safely.

## CRediT authorship contribution statement

**Chrysi Papalazarou:** Conceptualization, Methodology, Formal analysis, Writing – original draft. **Sima Qamhiyeh:** Conceptualization, Methodology, Writing – original draft. **Robert Kaatee:** Conceptualization, Methodology, Writing – original draft. **Joke De Rouck:** Conceptualization, Methodology, Formal analysis. **Esther Decabooter:** Conceptualization, Writing – review & editing. **Guido C. Hilgers:** Conceptualization, Writing – review & editing. **Koen Salvo:** Conceptualization. **Jacobus van Wingerden:** Conceptualization. **Hilde Bosmans:** Conceptualization. **Brent van der Heyden:** Conceptualization, Writing – review & editing. **Geert Pittomvils:** Conceptualization, Methodology, Resources. **Evelien Bogaert:** Conceptualization, Methodology, Formal analysis, Writing – review & editing, Supervision.

## Declaration of competing interest

The authors declare that they have no known competing financial interests or personal relationships that could have appeared to influence the work reported in this paper.
