# Supplementary material for: Survey on fan-beam computed tomography for radiotherapy: Current implementation and future perspectives of motion management and surface guidance devices
Source: Phys Imaging Radiat Oncol. 2023 Dec 10;29:100523. doi: 10.1016/j.phro.2023.100523 (PMC10767488; doi:10.1016/j.phro.2023.100523)
Supplement: Supplementary data 1 [file mmc1.pdf]

CT for Radiotherapy

Dear CT enthusiasts!

This survey has been setup in the framework of the NCS subcommittee on ***Quality Assurance of fan-beam CT for Radiotherapy*** consisting of MPE Medical Physicist Experts and MPA Medical Physicist Assistants from Belgium and the Netherlands. For more information on the subcommittee and its goals, please visit: https://radiationdosimetry.org/ncs/quality-assurance-of-fan-beam-ct-for-radiotherapy

The person(s) at your RT Radiotherapy department that is/are involved in CT-topics (commissioning, determination of the conversion curve, protocol optimization,...) will be most suited to fill in this survey. The survey covers the following topics:

- Institute and (Optional) Personal Information;
- CT-scanner Specifications;
- QA;
- Simulation Workflow;
- CT Imaging for Dose Calculation;
- Gated Imaging;
- Surface Guidance;
- Share your Vision.

Most probably a duo of an Medical Physicist Expert (MPE) and an Radiotherapy Technologist (RTT) who operates the CT-scanner daily, will get through the questions in the fastest way. The estimated time to fill in the survey is about **45 minutes**. We only need 1 answered survey per RT department.

Unfortunately, Microsoft Forms does not provide the ability to 'save and continue later on'. So once started, you need to complete the survey before closing it. We do apologize for this inconvenience. After submission, a pdf with your answers will be available for downloading.

We would be glad to receive your answer **before Monday, Oct 10th 2022.**

We do appreciate your contribution! Your response to this survey will help us to write a report with QA guidelines tailored to your needs. Thank you for your willingness and time!

On behalf of the NCS committee,

[names redacted]

Section 1

Institute and (Optional) Personal Information

All personal information (institute name, personal name, e-mailadress), given in this section will not be published, nor shared, without your acknowledgement and approval. This information can only be used internally in the NCS workgroup on 'Quality Assurance of fan-beam CT for Radiotherapy', and will be pseudonymized when opted for scientific communication of the study outcome.

1

Your institute is located in [Required question. One single answer allowed.]

The Netherlands

Belgium

the Grand Duchy of Luxembourg

2

OPTIONAL: Name of your institute [One line of text.].

Type your answer here

3

What is your profession? [Required question. One single answer allowed.]

Throughout this questionnaire we use specifications for 6 functions, that are described below and of which possible synonyms are formulated

1. **MPE Medical Physicist Expert Radiotherapy**
   *Klinisch Fysicus Radiotherapie RTKF (NL): person with recognition Stichting Opleiding Klinisch fysici - NL
   *Erkend Deskundige in de Medische Stralingsfysica - Radiotherapie / Expert agréé en Radiophysique
   Médicale (BE): person with recognition by FANC (Federaal Agentschap voor Nucleaire Controle - BE)
   *Expert en Physique Médicale en radiothérapie (DQPRM) (LUX)
2. **MPE Medical Physicist Expert Radiology**
   *idem see above but for domain radiology instead of domain radiotherapy
3. **MPA Medical Physicist Assistant**
   *Medical Physics Engineer
   *Linac technician
   *Assistent Medische Stralingsfysica; Assistant en Radiophysique Médicale (BE)
   *Klinisch Fysisch Medewerker (NL)
4. **RTT Radiotherapy Technologist**
   *Medisch Beeldvormings- en Bestralingsdeskundige (MBB); simulatie-verpleegkundigen
   *person who operates the CT-scanner in clinical workflow (selection of protocol, intialization of image reconstruction,....)
5. **RO Radiation Oncologist**
   *RT Physician
6. **PA Physician Assistant**
   *RT Assistant of the Physician, no clinical degree

MPE Radiotherapy

MPE Radiology

MPA

RTT

RO

PA

4

Do you want to be informed of the results of this survey? [Required question. One single answer allowed.]

Yes (please provide your personal details in question 5-7)

No

Section 2

Detailed Personal Information

5

First Name: [Required question. One line of text.]

Type your answer here

6

Surname: [Required question. One line of text.]

Type your answer here

7

E mail Address: [Required question. One line of text.]

Type your answer here

Section 3

CT-Scanners in your Department

8

How many CT scanners are used at your hospital to conduct radiotherapy treatment planning? [Required question. One single answer allowed.]

1

2

3

9

For how many CT scanner will you fill in this questionnaire (**max 3**)? [Required question. One line of text.]

Value must be a number

10

Please motivate your choice. [Required question. One single answer allowed.]

All of them

Only newest

Only CT-scanner in own RT department

Only one of more identical scanners

Section 4

CT Scanner Specifications: Scanner 1

Following questions need to be filled in per CT-scanner. It will be possible to fill in for another scanner at the end of this section.

11

Who is the manufacturer of your CT scanner? [Required question. One single answer allowed.]

GE

Philips

Siemens

Canon/Toshiba

12

What type of scanner do you have (Discovery, Go.sim, Brilliance,...)? [Required question. One line of text.]

Type your answer here

13

What is the number of simultaneous acquisition slices? [Required question. One single answer allowed.]

16

32

64

128

256

14

What is the bore size (cm)? [Required question. One line of text.]

Value must be a number

15

Where is the CT scanner for use in radiotherapy located? [Required question. One single answer allowed.]

Radiotherapy department

Radiology department

Other

16

The CT scanner is in clinical use since (yyyy-MM-dd)? [Required question. One line of text.]

Type your answer here

17

Image Acquisition: what tube voltage setting is used? [Required question. One single answer allowed.]

Fixed (120 kVp)

Fixed (other than 120 kVp)

2 different Fixed (120 kVp - 140 kVp)

Variable, patient optimized tube voltage

18

What reconstuction type is used (multiple answers possible)? [Required question. Multiple choice.].

Filtered Back Projection (FBP)

Iterative reconstruction (IR)

Artificial Intelligence based reconstruction (AI)

19

Do you apply a metal artefact correction algorithm? [Required question. One single answer allowed.]

No

Yes, always

Yes, when metal is present in the range of interest

Yes, but for some other indications/situations: please specify below

20

Do you apply a beam hardening correction algorithm? [Required question. One single answer allowed.]

No

Yes, always

Yes, but for some indications/situations: please specify below

21

What is the maximum standard acquisition FOV Field of View (cm) (not extendend)? [Required question. One line of text.]

Type your answer here

22

Do you use Extended FOV Field of View? [Required question. One single answer allowed.]

Yes

No

23

Do you use extended CT-number scale (16bit)? [Required question. One single answer allowed.]

Yes

No

24

Do you use contrast agents (in case ‘Other’, please specify)? [Required question. One single answer allowed.]

No

Yes, a fixed volume of contrast agent is used for adults and a fixed volume is used for children

Yes, a variable volume of contrast agent is used (e.g. according to age, size of the patient , kV tube voltage,...)

Section 5

CT-Scanners in your Department

25

Is there a second CT-scanner for which you want to give the specifications in this survey? [One single answer allowed.]

No

Yes

Section 6

CT Scanner Specifications: Scanner 2

Following questions need to be filled in per CT-scanner. It will be possible to fill in for another scanner at the end of this section.

26

Who is the manufacturer of your CT scanner? [Required question. One single answer allowed.]

GE

Philips

Siemens

Canon/Toshiba

27

What type of scanner do you have (Discovery, Go.sim, Brilliance,...)? [Required question. One line of text.]

Type your answer here

28

What is the number of simultaneous acquisition slices? [Required question. One single answer allowed.]

16

32

64

128

256

29

What is the bore size (cm)? [Required question. One line of text.]

Value must be a number

30

Where is the CT-scanner for use in radiotherapy located? [Required question. One single answer allowed.]

Radiotherapy department

Radiology department

31

The CT scanner is in clinical use since (yyyy-MM-dd)? [Required question. One line of text.]

Type your answer here

32

Image Acquisition: what tube voltage setting is used ? [Required question. One single answer allowed.]

Fixed (120 kVp)

Fixed (other than 120 kVp)

2 different fixed (e.g. 120 kVp - 140 kVp)

Variable, patient optimized tube voltage

33

What reconstruction type is used ? (multiple answers possible) [Required question. Multiple choice.].

Filtered Back Projection (FBP)

Iterative reconstruction (IC)

Artificial Intelligence based reconstruction (AI)

34

Do you apply a metal artefact correction algorithm?   [Required question. One single answer allowed.]

No

Yes, always

Yes, when metal is apparent in the range of interest

Yes, but for some other indications/situations: please specify below

35

Do you apply a beam hardening correction algorithm? [Required question. One single answer allowed.]

No

Yes, always

Yes but for some indications/situations: please specify below

36

Do you use Extended FOV Field of View? [Required question. One single answer allowed.]

Yes

No

37

Do you use extended CT-number scale (16bit)? [Required question. One single answer allowed.]

Yes

No

38

Do you use contrast agents (in case 'Other', please specify)? [Required question. One single answer allowed.]

No

Yes, a fixed volume of contrast agent is used for adults and a fixed volume is used for children

Yes, a variable volume of contrast agent is used (e.g. according to age, size of the patient , kV tube voltage,...)

Section 7

CT-Scanners in your Department

39

Is there a third CT-scanner for which you want to give the specifications in this survey?One single answer allowed.]

No

Yes

Section 8

CT Scanner Specifications: Scanner 3

Following questions need to be filled in per CT-scanner.

40

Who is the manufacturer of your CT scanner? [Required question. One single answer allowed.]

GE

Philips

Siemens

Canon/Toshiba

41

What type of scanner do you have (Discovery, Go.sim, Brilliance,...)? [Required question. One line of text.]

Type your answer here

42

What is the number of simultaneous acquisition slices? [Required question. One single answer allowed.]

16

32

64

128

256

43

What is the bore size (cm)? [Required question. One line of text.]

Value must be a number

44

Where is the CT scanner for use in radiotherapy located? [Required question. One single answer allowed.]

Radiotherapy department

Radiology department

45

The CT scanner is in clinical use since (yyyy-MM-dd)? [Required question. One line of text.]

Type your answer here

46

Image Acquisition: what tube voltage setting is used? [Required question. One single answer allowed.]

Fixed (120 kVp)

Fixed (other than 120 kVp)

2 different fixed (e.g. 120kVp - 140kVp)

Variable, patient optimized tube voltage

47

What reconstuction type is used (multiple answers possible)? [Required question. Multiple choice.].

FBP Filtered Back Projection

Iterative reconstruction

AI Artificial Intelligence based reconstruction

48

Do you apply a metal artefact correction algorithm? [Required question. One single answer allowed.]

No

Yes, always

Yes, when metal is apparent in the range of interest

Yes, but for some other indications/situations: please specify below

49

Do you apply a beam hardening correction algorithm? [Required question. One single answer allowed.]

No

Yes, always

Yes but for some indications/situations: please specify below

50

Do you use Extended FOV Field of View? [Required question. One single answer allowed.]

Yes

No

51

Do you use extended CT-number scale (16bit)? [Required question. One single answer allowed.]

Yes

No

52

Do you use contrast agents (in case 'Other', please specify)? [Required question. One single answer allowed.]

No

Yes, a fixed volume of contrast agent is used for adults and a fixed volume is used for children

Yes, a variable volume of contrast agent is used (e.g. according to age, size of the patient , kV tube voltage,...)

Section 9

Quality Assurance

Throughout this questionnaire we use specifications for 6 functions, that are described below and of which possible synonyms are formulated

1. **MPE Medical Physicist Expert Radiotherapy**
   *Klinisch Fysicus Radiotherapie RTKF (NL): person with recognition Stichting Opleiding Klinisch fysici - NL
   *Erkend Deskundige in de Medische Stralingsfysica - Radiotherapie / Expert agréé en Radiophysique
   Médicale (BE): person with recognition by FANC (Federaal Agentschap voor Nucleaire Controle - BE)
   *Expert en Physique Médicale en radiothérapie (DQPRM) (LUX)
2. **MPE Medical Physicist Expert Radiology**
   *idem see above but for domain radiology instead of domain radiotherapy
3. **MPA Medical Physicist Assistant**
   *Medical Physics Engineer
   *Linac technician
   *Assistent Medische Stralingsfysica; Assistant en Radiophysique Médicale (BE)
   *Klinisch Fysisch Medewerker (NL)
4. **RTT Radiotherapy Technologist**
   *Medisch Beeldvormings- en Bestralingsdeskundige (MBB); simulatie-verpleegkundigen
   *person who operates the CT-scanner in clinical workflow (selection of protocol, intialization of image reconstruction,....)
5. **RO Radiation Oncologist**
   *RT Physician
6. **PA Physician Assistant**
   *RT Assistant of the Physician, no clinical degree

53

Who performs / is involved in routine QA of the CT scanner? [Required question. Likert.

	For all tests	For some test or some levels	Never
1. MPE Radiotherapy			
2. MPE Radiology			
3. MPA			
4. RTT			
5. RO			
6. PA			

54

Have the following tests been included **during CT commissioning** ? [Required question. Likert.

	Yes	No
HU accuracy/stability		
Geometrical accuracy of image reconstruction		
Lasers (e.g. stability, distance to isocenter, mobile laser movement,...)		
Image quality (e.g. high contrast resolution, low contrast resolution, MTF...)		

	Yes	No
Dosimetry (e.g. CTDI CT Dose Index measurements, HVL Half Value Layer determination, ...)		
Connectivity or communication tests		
Table stability and movement accuracy checks		
End-to-end testing		
QA on extended FOV (e.g. HU accuracy, geometric accuracy)		
QA on extended CT number scale		
Tests on coordinate transformation		

55

Which tests are included in your **clinical routine testing**? [Required question. Likert.

	Daily	Weekly	Monthly	Halfyearly	Yearly	Not included
HU accuracy/stability						
Geometrical accuracy of image reconstruction						
Lasers (e.g. stability, distance to isocenter, mobile laser movement,...)						
Image quality (e.g. high contrast resolution, low contrast resolution, MTF...)						
Dosimetry (e.g. CTDI CT Dose Index measurements, HVL Half Value Layer determination, ...)						
Connectivity or communication tests						
Table stability and movement accuracy checks						
End-to-end testing						
QA on extended FOV (e.g. HU accuracy, geometric accuracy)						
QA on extended CT number scale						
Tests on coordinate transformation						

56

In case you perform other tests, beyond those in the previous list, please specify here, including their frequency. [Multiple lines of text.]

Type your answer here

Section 10

Simulation Workflow

57

Which POIs (Points of Interest) do you use?

· **Laser Origin**: Origin of the external lasers. Typically at the CT rotation axis, at some distance (e.g. 50cm) from the CT scan plane.

· **Localization Point**: Also known as 'Setup Reference Point' or 'Setup Origin'. This point is typically marked on the patient and used for initial setup of the patient for treatment. After this initial setup, the patient might be shifted to the plannings isocenter.

· **Planning Isocenter**: The isocenter of the treatment plan. During treatment the planning isocenter must be positioned at the isocenter of the treatment machine.
[Required question. Likert.

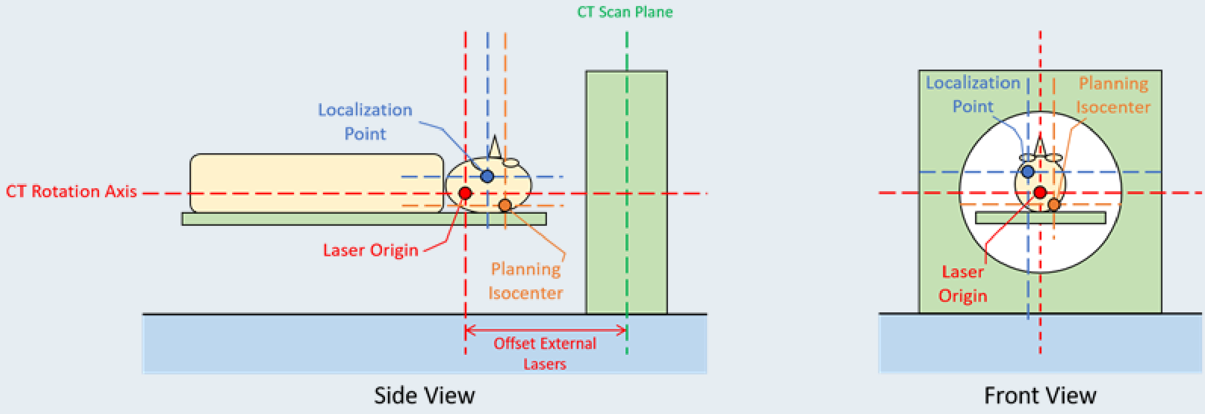

	**Mark on patient and on image data set**	**Mark on image data set**	**No [illegible]**
Laser Origin			
Localization Point			
Planning Isocenter			

58

Who defines the POIs? [Required question. Likert.

	**MPE on CT**	**RTT on CT**	**RO / PA on CT**	**MPE/MPA during treatment planning**	**RO/PA during treatment planning**	**Nobody, since not used**
Laser Origin						
Localization Point						
Planning Isocenter						

59

How is the **localization point** indicated (multiple answers possible, in case 'Other', please specify)? [Required question. Multiple choice.].

Localization point is not used

Opaque markers (ball bearings)

Tattoo Points

Lines and marks made by markers

60

Are **lasers** used to indicate the localization point (in case ‘Other’, please specify)? [Required question. One single answer allowed.]

No, no localization point is used

Yes, using fixed lasers

Yes, using movable lasers

61

How is the **planning isocenter** determined (multiple answers possible, in case ‘Other’, please specify)? [Required question. One single answer allowed.]

Planning Isocenter = Localization Point

Planning isocenter is determined per treatment

Section 11

CT Imaging for Dose Calculation

According to the different treatment techniques that you use (treatment with photons, protons, electrons or brachytherapy), specific questions will be asked

62

Do you use **SECT Single-Energy CT** imaging for **photon** planning?One single answer allowed.]

Yes

No

Section 12

SECT Imaging for Photon Planning

63

Which **TPS Treatment Planning System** do you use (multiple answers possible, in case ‘Other’, please specify)? [Required question. Multiple choice.].

RayStation

Eclipse

Monaco

Brainlab

Pinnacle

64

Which quantity is **reconstructed** by the CT system (multiple answers possible, in case ‘Other’, please specify)? [Required question. Multiple choice.].

CT-number [HU]

MD Mass Density [g/cm3] (e.g. Direct Density)

RED Relative Electron Density [-] (e.g. Direct Density)

65

Which quantity is **required by the TPS** for dose computation after CT conversion (multiple answers possible, in case 'Other', please specify)?
[Required question. Multiple choice.].

CT-number [HU]

MD Mass Density [g/cm3]

RED Relative Electron Density [-]

66

How did you define your **CT conversion curve**(s) (multiple answers possible, in case 'Other', please specify)?
[Required question. Multiple choice.].

Direct method: Use measured CT-numbers of tissue substitutes to define directly the conversion curve

Stoichiometric method: Use measured CT-numbers of tissue substitutes to calculate CT-numbers of biological tissues and use these calculated CT-numbers to define the conversion curve

Unit conversion: e.g. RED in HU-scale (RED water = 0 HU) to RED in physical scale (RED water = 1)

67

Which **phantom** do you use to determine the conversion curve(s) (multiple answers possible, in case 'Other', please specify)?

[Required question. Multiple choice.].

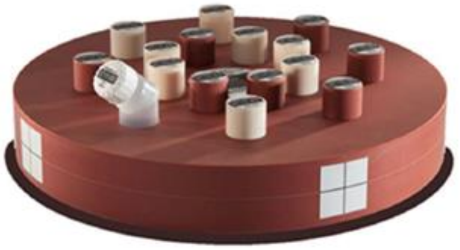 Gammex, Tissue Characterization Phantom, Model 467	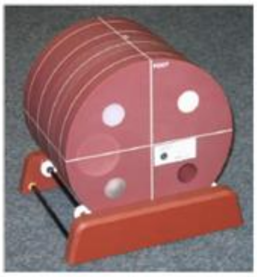 Gammex, RMI CT Phantom, Model 438	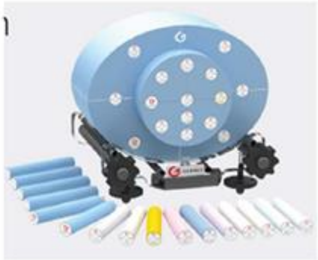 Sun Nuclear, Advanced Electron Density Phantom
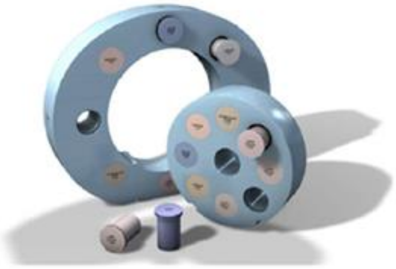 CIRS, Electron Density Phantom, Model 062M	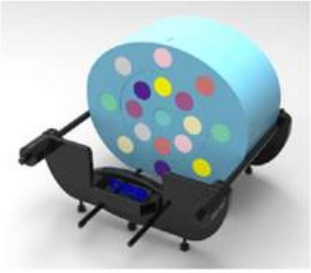 CIRS, Multi-Energy CT QA Phantom, Model 662	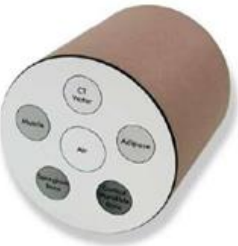 PTW, QRM Electron Density Phantom

Gammex: Tissue Characterization Phantom, Model 467

Gammex: RMI CT Phantom, Model 438

Sun Nuclear: Advanced Electron Density Phantom

CIRS: Electron Density Phantom, Model 062M

CIRS: Multi-Energy CT QA Phantom, Model 662

PTW: QRM Electron Density Phantom

The Phantom Laboratory: Catphan phantom

In-house made phantoms

No phantoms: we use a standard conversion curve

68

Do you use **metal inserts** in your phantom(s) (multiple answers possible)? [Required question. Multiple choice.].

Yes, for inclusion in the conversion curve

Yes, for metal artifact reduction checks

Yes, for differentiation of metallic implants

No

69

Do you use **multiple** conversion curves? (multiple answers possible, in case ‘Other’, please specify) [Required question. Multiple choice.].

Yes, per patient size (to account for beam hardening)

Yes, per CT-scanner: Type

Yes, per CT-scanner: SECT vs. DECT

Yes, per CT Acquisition: Tube voltage

Yes, per Image Reconstruction Algorithm

No, we use one supplied by the manufacturer

No, we use one in-house made conversion curve

Section 13

CT Imaging for Dose Calculation

According to the different treatment techniques that you use (treatment with photons, protons, electrons or brachytherapy), specific questions will be asked

70

Do you use **SECT** imaging for **proton** planning? One single answer allowed.]

Yes

No

Section 14

SECT Imaging for Proton Planning

71

Which **TPS Treatment Planning System** do you use (multiple answers possible, in case ‘Other’, please specify)? [Required question. Multiple choice.].

RayStation

Eclipse

72

Which quantity is **reconstructed** by the CT system ? (multiple answers possible, in case 'Other', please specify)? [Required question. Multiple choice.].

CT-number [HU]

SPR Stopping Power Ratio [-] (e.g. DirectSPR)

MD Mass density [g/cm3] (e.g. Direct Density)

RED Relative Electron Density [-] (e.g. Direct Density)

73

Which quantity is **required by the TPS** for dose computation after CT conversion? (multiple answers possible, in case 'Other', please specify)? [Required question. Multiple choice.].

CT-number [HU]

SPR Stopping Power Ratio [-]

MD Mass Density [g/cm3]

RED Relative Electron Density [-]

74

Do you use **other conversion methods** outside of the clinical CT / TPS for SPR estimation (in case 'Yes', please specify) ? [Required question. One single answer allowed.]

No

75

How did you define your **CT conversion curve**(s) (multiple answers possible, in case 'Other', please specify)? [Required question. Multiple choice.].

Direct method: Use measured CT-numbers of tissue substitutes to define directly the conversion curve

Stoichiometric method: Use measured CT-numbers of tissue substitutes to calculate CT-numbers of biological tissues and use these calculated CT-numbers to define the conversion curve

Unit conversion: e.g. SPR in HU-scale (SPR water = 0 HU) to SPR in physical scale (SPR water = 1)

76

Which **phantom** do you use to determine the conversion curve(s) (multiple answers possible)? [Required question. Multiple choice.].

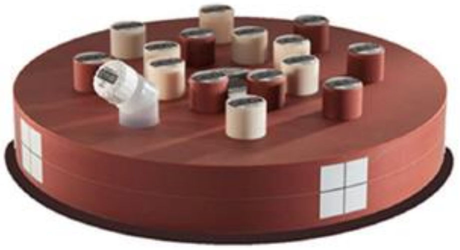<br>Gammex, Tissue Characterization Phantom, Model 467	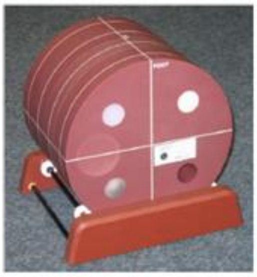<br>Gammex, RMI CT Phantom, Model 438	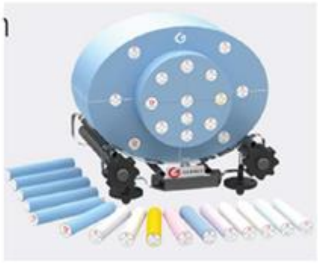<br>Sun Nuclear, Advanced Electron Density Phantom
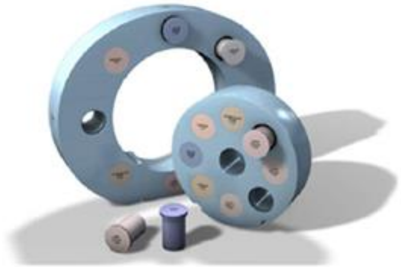<br>CIRS, Electron Density Phantom, Model 062M	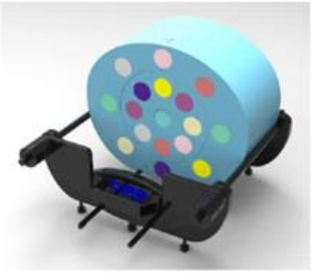<br>CIRS, Multi-Energy CT QA Phantom, Model 662	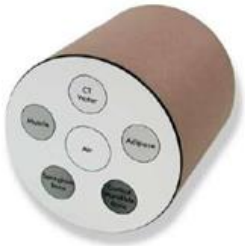<br>PTW, QRM Electron Density Phantom

Gammex: Tissue Characterization Phantom, Model 467

Gammex: RMI CT Phantom, Model 438

Sun Nuclear: Advanced Electron Density Phantom

CIRS: Electron Density Phantom, Model 062M

CIRS: Multi-Energy CT QA Phantom, Model 662

PTW: QRM Electron Density Phantom

The Phantom Laboratory: Catphan phantom

In-house made phantoms

No phantoms, we use a standard conversion curve

77

Do you use **metal inserts** in your phantom(s) (multiple answers possible)? [Required question. Multiple choice.].

Yes, for inclusion in the conversion curve

Yes, for metal artifact reduction checks

Yes, for differentiation of metallic implants

No

78

Do you use **multiple** conversion curves? (multiple answers possible, in case 'Other', please specify)? [Required question. Multiple choice.].

Yes, per patient size (to account for beam hardening)

Yes, per CT-scanner: Type

Yes, per CT-scanner: SECT vs. DECT

Yes, per CT Acquisition: Tube voltage

Yes, per Image Reconstruction Algorithm

No, we use one supplied by a manufacturer

No, we use one in-house made conversion curve

Section 15

CT Imaging for Dose Calculation

According to the different treatment techniques that you use (treatment with photons, protons, electrons or brachytherapy), specific questions will be asked

79

Do you use **DECT Dual-Energy CT** imaging for **photon** planning? One single answer allowed.]

Yes

No

Section 16

DECT Imaging for Photon Planning

80

Which **TPS Treatment Planning System** do you use (multiple answers possible, in case 'Other', please specify)? [Required question. Multiple choice.].

RayStation

Eclipse

Monaco

Brainlab

Pinnacle

81

Which quantity is **reconstructed** by the CT system (multiple answers possible, in case 'Other', please specify)? [Required question. Multiple choice.].

CT-number [HU]

MD Mass Density [g/cm3] (e.g. Direct Density, Dual-Energy CT conversion by firmware)

RED Relative Electron Density [-] (e.g. Direct Density, Dual-Energy CT conversion by firmware)

82

Which quantity is **required by the TPS** for dose computation after CT conversion (multiple answers possible, in case ‘Other’, please specify)?
[Required question. Multiple choice.].

CT-number [HU]

MD Mass Density [g/cm3]

RED Relative Electron Density [-]

83

For which **treatment sites** do you use DECT for dose computation (multiple answers possible, in case ‘Other’, please specify)? [Required question. Multiple choice.].

Neuro

Head and neck

Breast

Lung

Lymphoma

Oesophagus

Abdomen

Pelvis

Extremities

Pediatric

84

Do you use a **dedicated DECT conversion curve** (multiple answers possible, in case ‘Other’, please specify)? [Required question. Multiple choice.].

Yes

No, we use our SECT conversion curve

85

Do you perform **additional QA** measurements on DECT images (multiple answers possible, in case ‘Other’, please specify)? [Required question. Multiple choice.].

CT-number Stability: for every energy

CT-number Stability: for virtual monoenergetic images, relative electron density images, mass density images, ...

Image Quality: for every energy

Image Quality: for virtual monoenergetic images, relative electron density images, mass density images, ...

86

Which **phantom** do you use to determine the conversion curve(s) (multiple answers possible, in case 'Other', please specify)? [Required question. Multiple choice.].

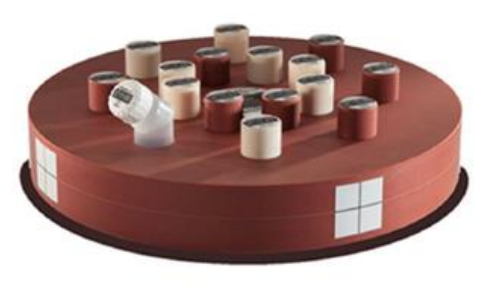 Gammex, Tissue Characterization Phantom, Model 467	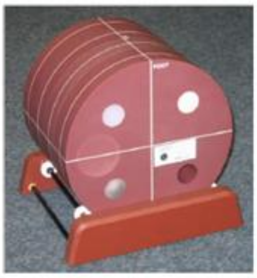 Gammex, RMI CT Phantom, Model 438	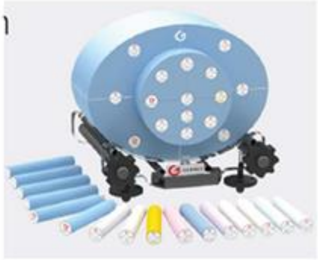 Sun Nuclear, Advanced Electron Density Phantom
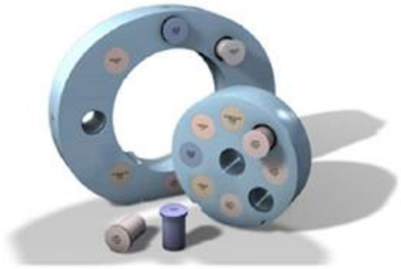 CIRS, Electron Density Phantom, Model 062M	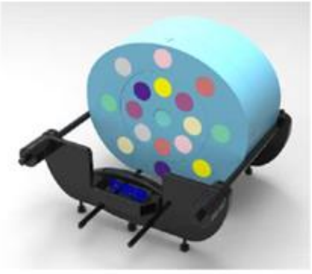 CIRS, Multi-Energy CT QA Phantom, Model 662	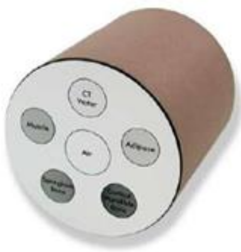 PTW, QRM Electron Density Phantom

Gammex: Tissue Characterization Phantom, Model 467

Gammex: RMI CT Phantom, Model 438

Sun Nuclear: Advanced Electron Density Phantom

CIRS: Electron Density Phantom, Model 062M

CIRS: Multi-Energy CT QA Phantom, Model 662

PTW: QRM Electron Density Phantom

The Phantom Laboratory: Catphan phantom

In-house made phantoms

No phantoms, we use a standard conversion curve

87

Do you use **metal inserts** in your phantom(s) (multiple answers possible, in case 'Other', please specify)? [Required question. Multiple choice.].

Yes, for inclusion in the conversion curve

Yes, for metal artifact reduction checks

Yes, for differentiation of metallic implants

No

88

Do you use **multiple** conversion curves? (multiple answers possible, in case 'Other', please specify) [Required question. Multiple choice.].

Yes, per patient size (to account for beam hardening)

Yes, per CT-scanner: Type

Yes, per CT-scanner: SECT vs. DECT

Yes, per CT Acquisition: Tube voltage

Yes, per Image Reconstruction Algorithm

No, we use one supplied by a manufacturer

No, we use one in-house made conversion curve

Section 17

CT Imaging for Dose Calculation

According to the different treatment techniques that you use (treatment with photons, protons, electrons or brachytherapy), specific questions will be asked

89

Do you use **DECT** imaging for **proton** planning?One single answer allowed.]

Yes

No

Section 18

DECT for Proton Planning

90

Which **TPS Treatment Planning System** do you use (multiple answers possible, in case 'Other', please specify)? [Required question. Multiple choice.].

RayStation

Eclipse

91

Which quantity is **reconstructed** by the CT system (multiple answers possible, in case 'Other', please specify) ?
[Required question. Multiple choice.].

CT-number [HU]

SPR Stopping Power Ratio [-] (e.g. DirectSPR)

MD Mass Density [g/cm3] (e.g. Direct Density, Dual-Energy CT conversion by firmware)

RED Relative Electron Density [-] (e.g. Direct Density, Dual-Energy CT conversion by firmware)

92

Which quantity is **required by the TPS** for dose computation after CT conversion (multiple answers possible, in case 'Other', please specify) ?
[Required question. Multiple choice.].

CT-number [HU]

SPR Stopping Power Ratio [-]

MD Mass Density [g/cm3]

RED Relative Electron Density [-]

93

Do you use **other conversion methods** outside of the clinical CT / TPS for SPR estimation (in case 'Yes', please specify)? [Required question. One single answer allowed.]

No

94

For which **treatments sites** do you use DECT for dose computation (multiple answers possible, in case ‘Other’, please specify)?
[Required question. Multiple choice.].

Neuro

Head and neck

Breast

Lung

Lymphoma

Oesophagus

Abdomen

Pelvis

Extremities

Pediatric

95

How do you use **DECT** for dose computation? (multiple answers possible, in case 'Other', please specify)? [Required question. Multiple choice.].

Virtual monoenergetic images in combination with the corresponding SECT conversion curve

Virtual monoenergetic images in combination with a DECT specific conversion curve

Combining high and low kV images to estimate SPR Stopping Power Ratios

96

Do you perform **additional QA** measurement on DECT images (multiple answers possible, in case 'Other', please specify)?
[Required question. Multiple choice.].

CT-number stability: for every energy

CT-number stability: for virtual monoenergetic images, relative electron density images, mass density images, SPR images, ...

Image quality: for every energy

Image quality: for virtual monoenergetic images, relative electron density images, mass density images, SPR images, ...

97

Have you reduced your **range uncertainty** margins in the treatment planning when implementing DECT (in case 'Other', please specify)?
[Required question. One single answer allowed.]

No, we use the same range uncertainty margin for SECT and DECT

98

Which **phantom** do you use to determine the conversion curve(s) (multiple answers possible, in case 'Other', please specify)?
[Required question. Multiple choice.].

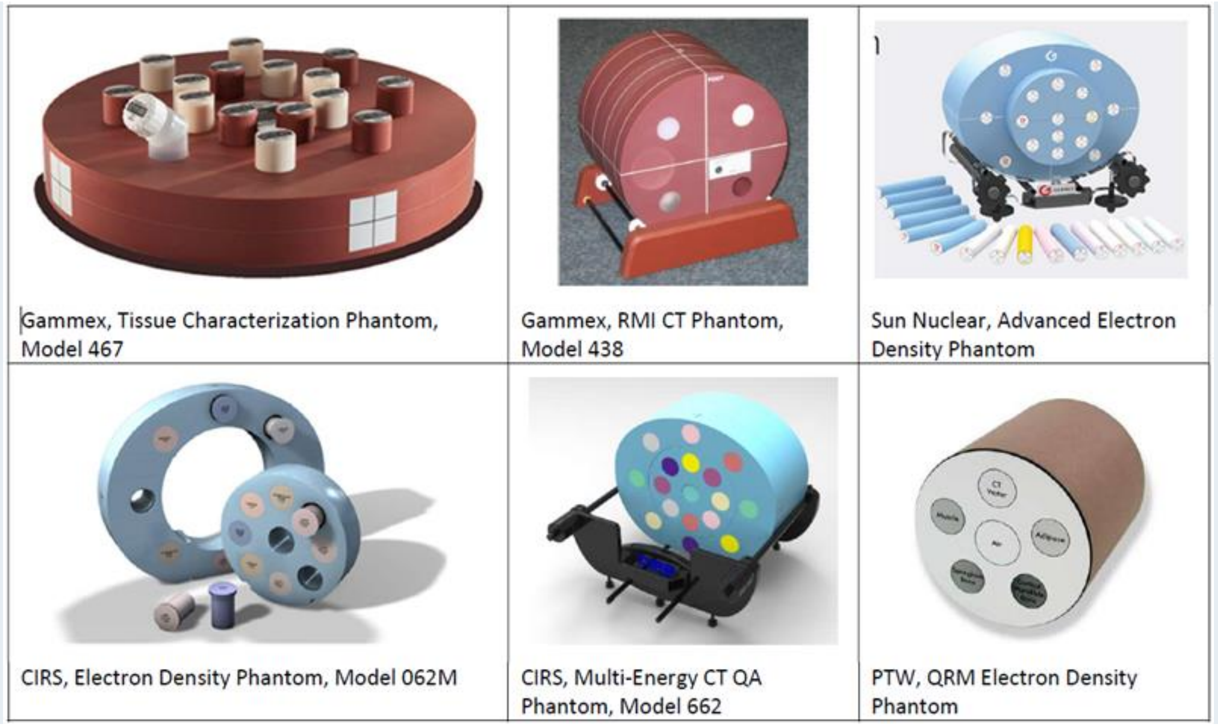

Gammex: Tissue Characterization Phantom, Model 467

Gammex: RMI CT Phantom, Model 438

Sun Nuclear: Advanced Electron Density Phantom

CIRS: Electron Density Phantom, Model 062M

CIRS: Multi-Energy CT QA Phantom, Model 662

PTW: QRM Electron Density Phantom

The Phantom Laboratory: Catphan phantom

In-house made phantoms

No phantoms, we use a standard conversion curve

99

Do you use **metal inserts** in your phantom(s) (multiple answers possible, in case ‘Other’, please specify)? [Required question. Multiple choice.].

Yes, for inclusion in the conversion curve

Yes, for metal artifact reduction checks

Yes, for differentiation of metallic implants

No

100

Do you use **multiple** conversion curves (multiple answers possible, in case 'Other', please specify)? [Required question. Multiple choice.].

Yes, per patient size (to account for beam hardening)

Yes, per CT-scanner: Type

Yes, per CT-scanner: SECT vs. DECT

Yes, per CT Acquisition: Tube voltage

Yes, per Image Reconstruction Algorithm

No, we use one supplied by a manufacturer

No, we use one in-house made conversion curve

Section 19

CT Imaging for **Electron** Dose Caclulation

According to the different treatment techniques that you use (treatment with photons, protons, electrons or brachytherapy), specific questions will be asked

101

Do you use CT imaging for **electron** dose calculation?One single answer allowed.]

Yes

No

Section 20

CT Imaging for Electron Planning

102

Which **TPS Treatment Planning System** do you use (multiple answers possible, in case ‘Other’, please specify)? [Required question. Multiple choice.].

RayStation

Eclipse

Pinnacle

Monaco

103

Do you use dedicated image acquisition or reconstruction for electron planning purposes? (multiple answers possible, in case ‘Other’, please specify) [Required question. Multiple choice.].

No

Yes, we make sure that reconstruction slice thickness is small enough

Yes, we use other reconstruction kernels

Yes, we have other guidelines for FOV field of view (smaller/larger)

104

Do you use a dedicated conversion curve for electron planning? In case 'yes', please specify. [Required question. One single answer allowed.]

No, we use the same as for photon planning

Section 21

CT Imaging for **Brachytherapy** Dose Caclulation

According to the different treatment techniques that you use (treatment with photons, protons, electrons or brachytherapy), specific questions will be asked

105

Do you use CT imaging for **brachytherapy** dose calculation or seed verification?One single answer allowed.]

Yes

No

Section 22

CT Imaging for Brachytherapy Planning

106

Which **TPS Treatment Planning System** do you use for dose calculation?One single answer allowed.]

Oncentra

RayStation

BrachyVision

107

CT imaging is used for brachytherapy for... (multiple answers possible, in case 'Other', please specify) [Required question. Multiple choice.].

Dose calculation

Seed implant verification (e.g. prostate)

Applicator position verification (e.g. cervix)

108

Do you use dedicated image acquisition or reconstruction for brachytherapy purposes? (multiple answers possible, in case 'Other', please specify) [Required question. Multiple choice.].

No

Yes, we make sure that reconstruction slice thickness is small enough

Yes, we use other reconstruction kernels

Yes, we have other guidelines for FOV field of view (e.g. smaller)

Yes, we use low dose scan for applicator or seed position verifcation

109

Does your Treatment Planning System for brachytherapy take tissue heterogeneity into account? [Required question. One single answer allowed.]

No

Yes

Section 23

Gated Imaging

110

Do you use gated imaging (meaning breathing correlated 4DCT or breath-hold) in the clinic ? [Required question. One single answer allowed.]

Yes

No

Section 24

Gated Imaging: Specifications

With gated imaging we mean breathing correlated 4DCT or breath-hold

111

Which device is used to record the surrogate breathing signal for gated imaging (in case 'Other', please specify)? [Required question. Multiple choice.].

Anzai Belt

Vision RT Surface Guidance

CRAD Sentinel Surface Guidance

Varian RGSC

Varian RPM

Internal imaging (e.g. movenement of diaphrgm)

Spirometer

Philips bellows

112

Which type of reconstrution method is used for gated imaging (multiple answers possible, in case 'Other', please specify)? [Required question. Multiple choice.].

Retrospective 4DCT - amplitude based binning

Retrospective 4DCT - phase based binning

Prospective 4DCT amplitude based binning

Prospective 4DCT phase based binning

DIB Deep inspiration breath-hold

113

How many bins do you reconstruct ? [Required question. One line of text.]

Value must be a number

114

Which software do you use for the image reconstruction of 4DCT (e.g. bins, average, MIP, mid-ventilation...) (multiple answers possible)? [Required question. Multiple choice.].

CT software

Third party software that is not your TPS (please specify in next question)

In house developed software (please specify in next question)

TPS

115

In case you use third party software for 4DCT image reconstruction, please specify below the vendor and name. [Multiple lines of text.]

Type your answer here

116

In case you use in house developed software for 4DCT image reconstruction, please specify briefly why and its functionality [Multiple lines of text.]

Type your answer here

117

The 4DCT reconstructed images of patients are... [Required question. One single answer allowed.]

Always and systematically inspected when patient is still on the CT simulation couch

Always and systematically inspected; a positive evaluation of the images allows the patient to leave the department

Always and systematically inspected after the patient has left the departement

Inspected but not systematically (no specific protocol and/or no fixed defined timepoint)

118

What kind of inspection do you perform on the 4DCT reconstruction of each patient (multiple answers possible, in case 'Other', please specify)? [Required question. Multiple choice.].

Appearance of stack artefacts

Apprearane of interpolation artefacts

HU value of average reconstruction vs HU of bins

Visual evaluation of breathing curve during acquisition

Visual evaluation of bins in video-mode

Visual inspection of lung lobe edges continuity

Visual inspection of skin contour

119

How is image quality judged to be insufficient (multiple answers possible, in case 'Other', please specify)? [Required question. Multiple choice.].

A protocol that defines when the 4DCT image quality is insufficient and the actions to be taken has been setup

No such protocol has been setup, but RTTs are trained to judge themselves and call for help when doubting

MPE always evaluates image quality (for each patient)

RO always evaluates image quality (for each patient)

120

For which indications do you use gated imaging and what type? (Select the most commonly used) [Required question. Likert.]

	**4DCT with abdominal compression**	**4DCT without abdominal compression**	**Breath-hold (inspiration, expiration, shallow)**	**Other**	**No gated imaging**
Lung					
Liver					
Pancreas					
Stomach					
Spleen					
Adrenal					
Breast (left sided)					
Breast (right sided)					

121

Do you use gated imaging for other sites than specified in the previous question? If 'Yes', please specify here. [Multiple lines of text.]

Type your answer here

122

Do you mark your patient during gated imaging? (in case 'Yes, please specify below) [Required question. One single answer allowed.]

No

123

Have the following QA tests regarding gated imaging been included **during commissioning**? [Required question. Likert.

Calibration check of gating system
HU consistency over bins and average or other recons
Contour consistency over bins and average or other recons
Amplitude accuracy (comparing amplitudes of moving and steerable phantom with the amplitude in the resulting images),
Geometric accuracy of non moving objects (e.g. in phantom) in bins and average or other recons,
Couch sag (e.g. in case wall or ceiling mounted systems)

124

Are there other tests than stated in the question above, that you perform during **commisionning** for gated imaging ? If 'Yes', please specify. [Multiple lines of text.]

Type your answer here

125

Which tests regarding gated imaging are included in **routine clinical testing**? [Required question. Likert.]

	**Daily**	**Weekly**	**Monthly**	**Halfyearly**	**Yearly**	**Not included**
Calibration check of gating system						
HU consistency over bins and average or other recons						
Contour consistency over bins and average or other recons						
Amplitude accuracy (comparing amplitudes of moving and steerable phantom with the amplitude in the resulting images),						
Geometric accuracy of non moving objects (e.g. in phantom) in bins and average or other recons,						
Couch sag (e.g. in case wall or ceiling mounted systems)						

126

Who is perfoming the QA tests regarding gated imaging? [Required question. Likert.

	MPE Radiotherapy	MPE Radiology	MPA	RTT	RO	PA	Nobody
Calibration check of gating system							
HU consistency over bins and average or other recons							
Contour consistency over bins and average or other recons							
Amplitude accuracy (comparing amplituds of moving and steerable phantom with the amplitude in the resulting images),							
Geometric accuracy of non moving objects (e.g. in phantom) in bins and average or other recons,							
Couch sag (e.g. in case wall or ceiling mounted systems)							

127

Are there other tests than stated in the questions above, that you perform during **routine clinical testing** for gated imaging ? If 'Yes', please specify the task(s), who is peforming the task(s) and the frequency of testing. [Multiple lines of text.]

Type your answer here

Section 25

Surface Guidance

128

Is a surface guidance implemented in the CT-simulator room, in your department? [Required question. One single answer allowed.]

Yes

No

Section 26

Surface Guidance Specifications

129

Who is the manufacturer of your surface guidance system (in case 'Other', please specify)? [Required question. One single answer allowed.]

C-RAD

Vision-RT

Varian

Brainlab

130

The surface guidance system installed in the CT-room is used for... [Required question. [Likert.]

	Now	In the near future (< 1-2 year)	In the far futu re (5 years)	Not in the scope of implementatio n at our clinic
Determination of the reference surface for patient positioning				
Capturing of the breathing sign al for 4DCT imaging				
Capturing of the breathing sign al for breath-hold				
Collision prediction (between patient and treatment unit)				
Other, please specify in the next question				

131

In case the surface guidance system in the CT-room for simulation is used for other purposes than stated in the question above, please specify. [Multiple lines of text.]

Type your answer here

132

Did you feel the need to take lighting conditions in the CT-room into account when installing and using the surface guidance system at simulation? [Required question. One single answer allowed.]

No because the surface guidance system used is not sensitive to ambient lighting conditions

Yes, we generate the same lighting conditions during clinical flow and QA, as used during camera calibration

No

133

What type of phantom(s) do you use for QA of the surface guidance system at the level of simulation (multiple answers possible, in case 'Other', please specify)? [Required question. Multiple choice.].

Static, geometrical accurate phantom

Static, ‘irregular surface’ or anatomy-like phantom for surface detectability and reproducibility tests

Dynamic phantom for surface guidance-based motion management strategy testing

Phantoms in different light and dark tones for responsiveness testing of the camera on different skin tones

134

Are your phantoms for testing the surface guidance system at simulation level (multiple answers possible)... [Required question. Multiple choice.].

Bought

In house made

135

The phantom(s) I use for testing the surface guidance system at the level of simulation are ... [Required question. One single answer allowed.]

All the same as the one(s) used on the linac

All specific for CT

Some are the same and some are CT specific or linac specific

136

Please specify particular phantoms for testing the surface guidance system at CT-simulation level. [Multiple lines of text.]

Type your answer here

137

In case you use your surface guidance system as a QA tool for your CT-scanner (like couch mechanical motion check or other) please specify . [Multiple lines of text.]

Type your answer here

Section 27

Share Your Vision

138

According to your opinion, what techniques should be additionally implemented in your clinical workflow? Please order according to importance (most important first). [Classification.]

DECT for delineation purposes
DECT for calculation purposes
Breathing related 4DCT
Surface guidance in the CT-room
Tattooless workflow
Photon counting system

139

Which topic(s) deserve more attention / budget in the future, in general? Please order according to importance (most important first). [Classification].

Image quality in CT for radiotherpy, together with optimizing of CT scan protocols
Broad detector size enabling fast scanning
Dual energy scanning for visualization
Dual energy scanning for dose calculation
CT-imaging dose with registration in a dose-management-system; dose optimization
More or specialised training for operation/ commissioning of CT scanner
Gated imaging (4DCT, breath-hold)

Tattooless workflow

140

In the future spectral imaging (e.g. by DECT) will be the standard for CT-scanners in radiotherapy, at least for some indications [Required question. Net Promoter Score.]

I do not agree

I agree

141

CT scanning for radiotherapy has evolved a lot in the last decade. In order to cover the increased complexity, I consider the following actions necessary in the radiotherapy domain. Please order according to importance (most important first). [Classification.]

Additional and dedicated training
Collaboration with MPE Radiology and/or Radiologists
Support from skilled personnel of vendor
Clear guidelines for CT for RT
Time to get more acqainted with the existing literature

142

I am worried about the exposure burden to the patient related to CT-scans taken during radiotherapy e.g. for adaptive radiotherapy, 4DCT, full-empty bladder, free-breathing and DIB, ... [Required question. One single answer allowed.]

Yes

No

143

Please formulate here any thougths that you want to share with us. [Multiple lines of text.]

Type your answer here

Section 28

The end of the survey, but not the end of the story!

You have reached the end of this survey!

We are very gratefull for your contribution and thank you for your willingness and time!
In case you have entered your email address for feedback of the results, you will be contacted.

More information about the continuation of our work can be found by contacting one of the members, or on the NCS website.

Kind regards,

The NCS subcommittee on Quality Assurance of fan-beam CT for Radiotherapy
