# Supplementary material for: Survey on fan-beam computed tomography for radiotherapy: Current implementation and future perspectives of motion management and surface guidance devices
Source: Phys Imaging Radiat Oncol. 2023 Dec 10;29:100523. doi: 10.1016/j.phro.2023.100523 (PMC10767488; doi:10.1016/j.phro.2023.100523)
Supplement: Supplementary data 2 [file mmc2.docx]

In this supplement, we provide some additional information and clarification on specific topics that could not be covered in detail in the main manuscript.

# Surface guidance for simulation

The current and future applications of Surface Imaging (SI), as reported by the survey respondents, are summarized below. Percentages below are expressed as a fraction of the respondents who indicated using SI (N=10).

 
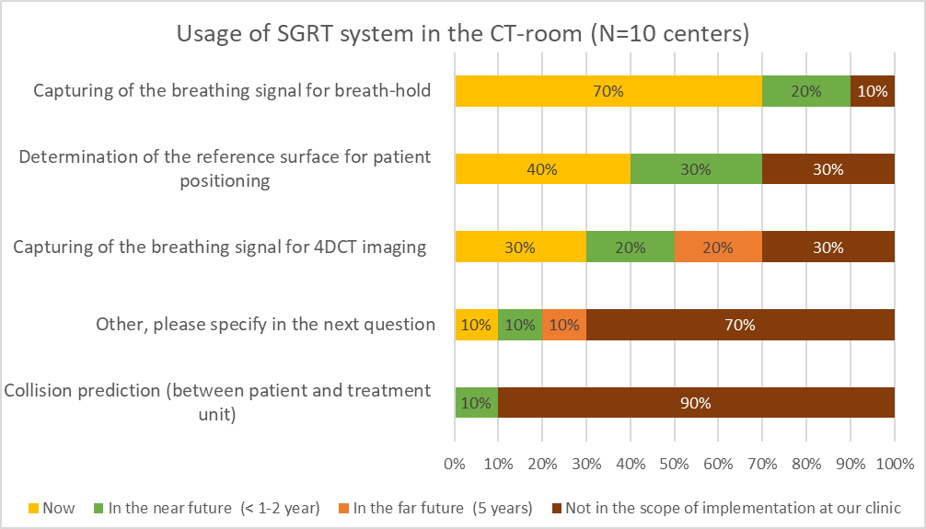


Figure 1 - Current and future applications of surface imaging, as reported in the survey.

The majority of SI users responding to the survey use the system for capturing the breathing signal for breath-hold. This technique is well established by 70% of the respondents and a further 30% are also interested in implementing in the near future. Regarding the use of surface imaging for the creation of a reference surface, the survey question explicitly asked whether the SI system installed in the CT room is used for determination of a reference surface for patient positioning. This means a reference surface directly based on the imaged surface and not deducted from the skin-contour on a DICOM CT image set. This is practiced by 40% of the respondents, while 30% of the respondents wish to implement this technique in the near or far future.

The survey did not explore the technical difficulties which hindered some of the respondents from implementing the technology at the time of the survey. This was beyond the scope of this survey. Nonetheless, private communication with the different respondents and key technology providers suggests that the technical features are available for such an application, as 40% of the respondents already do use this technique. However, the installations are very site specific. Additionally, the motion pattern of the CT couch is different from that during the treatment. Furthermore, only a few providers can offer a DICOM format of the patient surface and this further complicates data transition between the different oncology systems.

Capturing of the breathing signal for 4DCT acquisitions was performed by 30% of the respondents. Again, the survey did not explore the technical difficulties which hindered some of the further respondents from implementing the capture in clinical practice.

Three respondents chose the option “Other” and listed as additional applications: research (one respondent), tracking of the used accessories/patient support items and positioning (two respondents).
